# Omitted technical measures: establishing evidence for the necessity of problem formulation and validation in system alternative selection

**DOI:** 10.1007/s00163-026-00488-y

**Published:** 2026-06-24

**Authors:** Casey E. Eaton, Kelly X. Campo, August Longhurst, Bryan Mesmer

**Affiliations:** 1 Industrial and Systems Engineering, Auburn University, Auburn, AL USA; 2 Industrial & Systems Engineering and Engineering Management Department, The University of Alabama in Huntsville, Huntsville, AL USA

**Keywords:** Technical measurement, Decision making, Measurement, Contract award, System acquisition

## Abstract

Design and acquisition of large-scale complex engineered systems can use technical measures to compare system alternatives through setting constraints on those measures and/or providing objectives using the measures. Selecting a technical measure set can be uncertain, with little selection guidance available, and difficult to validate, potentially leading to omitting technical measures. This research examines the impact of omitting technical measures on system alternative selection using a case study of real-world technical measures and system alternatives. A requirements-based constraint framework and an optimization-based objective function framework are developed using a set of real-world technical measures. The research models how omissions of technical measures lead to choosing a different system alternative. The impacts are demonstrated through an application of the NASA Human Landing System (HLS) using 13 system alternatives, including the systems proposed to NASA by Blue Origin, SpaceX, and Dynetics. The research finds that omissions in the constraint framework open the design space, potentially changing the system alternative chosen. Omissions in the objective function framework alter the indicated ordinal preference for the system alternatives, changing the system alternative chosen. An omitted technical measure on the side of the acquirer may change the system alternative selected, directing millions of dollars towards a specific organization and system. This research highlights the practical impacts of omissions of technical measures for awarding contracts during system acquisition. The likelihood of omissions, the difficulty to validate outcomes, and the impacts of omissions demonstrated in this research form evidence that the connection amongst problem formulation, problem solving, and validation must be emphasized if used for system alternative selection.

## Introduction

Large-scale complex engineered systems such as submarines, power systems, satellites, or nuclear power plants take significant effort to design and deploy due to scale, cost, and complexity (Bloebaum and McGowan 2012). One key informant in current practice when selecting amongst system alternatives is technical measure sets. Measurement enables a complex system alternative to be evaluated by individual measurable attributes which are intended to aid in understanding the overall system design. While individual technical measures are attributes of the system, the set of technical measures intends to assess the overall preference for a system. If the amalgamation (by whatever method) of individual measures is superior for one system, in current practice, it can be interpreted that the system is preferred. System alternative selection built on technical measures depends on the set of measures selected. The assumption and implications of a set of technical measures being complete is investigated in this research.

### Research objective

To provide a realistic assessment of current measurement practice in system selection during the acquisition process, two common frameworks are modeled to determine the impacts of technical measure omissions on system alternative selection. The following research question addresses the omission of technical measures as used in a constraint framework and an objective function framework: “*What impacts can omissions of technical measures have on system alternative selection when the decision process is performed using a constraint framework and under an objective function framework?”*

Modeling the two frameworks demonstrates measurement challenges in system acquisition: the impact of omitted technical measures and the impact of the measurement framework. The implications of omissions in each framework are compared and paths for improving measurement practice in system acquisition are discussed, emphasizing the need for connecting frameworks developed to problem formulation and validation.

### Background

The background section describes the considerations for system acquisition, how technical measurement is used in systems design, the constraint framework, the objective framework, how the two frameworks compare, prior research regarding the impact and management of omitted measures, and a discussion of why measures may be omitted in the systems engineering and design domain.

#### System acquisition process and unique considerations

System acquisition is the process of one organization (often referred to as the “acquirer”) developing a system in partnership with another organization (often referred to as the “supplier”). Often in system acquisition, the acquirer is a government organization which selects among different system alternatives proposed by suppliers (often called “contractors”). System acquisition is often competitive (*Federal Acquisition Regulations System*, 2026; *NASA Federal Acquisition Regulation Supplement*, 2026), introducing the need to select a system alternative from those provided by competing suppliers. The process often involves communication of preferences for the system and evaluations of the system alternatives proposed. System alternatives can be at multiple stages of development, such as completed systems or proposed system concepts.

While individuals as consumers often weigh different measurements of a purchase implicitly, system acquisition faces different pressures. Stakeholders responsible for system alternative selection may not be able to intuitively weigh a set of technical measures that span multiple domains. Additional scrutiny over contract awards makes the set of technical measures critical for either government or private industry. The large-scale complex engineered systems that are typically acquired involve multiple subsystems, cross many domains, and require many experts to develop. In turn, the evaluation and selection amongst these system alternatives is likewise complex. However, government acquisition processes seek to minimize costs associated with the process (*Federal Acquisition Regulations System*, 2026) and can be subject to short timelines for rapid acquisitions (*Middle-Tier Defense Acquisitions*, 2023), potentially limiting the extent of an analysis that can be performed.

Government system acquisition has the additional concern of providing evidence for such major public decisions. Awards can be subject to protests such as in the case of the NASA Human Landing System (Cowing, 2021; U. S. Government Accountability Office 2021b). Suppliers and the public have a vested interest in these contracts which can bring significant funding, employment, and prestige to the chosen organization. The Federal Acquisition Regulation includes references to “integrity, fairness, and openness” (*Federal Acquisition Regulations System*, 2026). Evaluation of measures across system alternatives provides one justification that the decisions made were consistent and transparent.

#### Technical measurement in systems engineering and design

Large-scale complex engineered systems can be understood by assessing individual attributes numerically through measures. For such systems which can cost billions of dollars and take many years to develop, it is critical to select the preferred system alternative. Technical measures are intended to provide a clear and consistent decision-making tool. As observed by (Ferguson & Bryden, 2026), in practice, engineering decisions are expected to structured, transparent, and explainable. Technical measurement can replace or aid other concept selection practices such as meetings, voting, a single stakeholder choosing, or eliciting stakeholder preferences which are described by (Toh et al. 2015). These concept selection practices may not be able to document clear justification for decisions. Technical measures are often the basis for formal concept selection methods, where decision makers seek to determine which system alternatives (concepts) fulfill the “decision making criteria” (Toh and Miller 2016), or in this paper’s terminology, technical measures.

Technical measures interact amongst each other and influence a system *as a set*. According to guidance in systems engineering and design literature, sets of technical measures are supposed to be “complete” (Mackley 2005; Sahraoui 2013; Systems Engineering Measurement Primer, 1998), meaning not missing relevant measures. However, achieving completeness may be difficult, particularly for novel systems, for which technical measure sets may not be able to be based on prior systems. Previous research has observed that omitting technical measures can result in undesired systems (Eaton and White 2021; Mackley 2005). It has been observed that publicly available guidance for selecting technical measure sets may be contradictory (Eaton et al. 2024), suggesting that practitioners may not easily be able to select a complete set. Technical measures can broadly be applied in two frameworks: constraint frameworks or objective function frameworks.

#### Frameworks and design information

In this research, constraint and objective function frameworks are used to describe two main approaches to measurement in systems design. While different frameworks could exist outside of constraint and objective function, such as random choice, the constraint and objective framework appear consistently in systems engineering and systems design literature in practice (Collopy and Hollingsworth 2009; *NASA Systems Engineering Handbook*, 2019) and research (Collopy and Hollingsworth 2009; Hupman et al. 2015; Murugaiyan et al. 2016; Vermillion and Malak 2020; Vrolijk and Szajnfarber 2023). Frameworks, as used in this research, “guide the individuals’ decisions to result in the best overall design in the eyes of the stakeholder” (Hupman et al. 2015).

Frameworks, as used in this research, frame information in a systems design or selection problem. The same set of technical measures can be used in many ways; in this research the same set of technical measures is framed through a constraint framework and an objective function framework to explore the impacts of omitted measures within each framework. Prior research has identified that representation choices can impact a system’s perceived modularity and complexity (Hennig and Szajnfarber 2023). While this research focuses on demonstrating the impacts mathematically, framing has been shown to have significant impacts on human behavior and choice (Carpenter 2018). How information is framed may have a significant impact on design choices, both through mathematical impacts in formal approaches and through cognition impacts. This research examines only the mathematical impacts from the framework itself without evaluating potential cognitive or behavioral impacts.

**Fig. 1:**
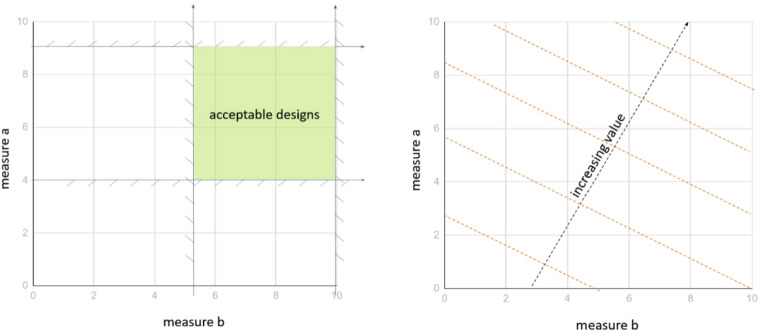
Constraint framework (left) and objective function framework (right)

For the two frameworks discussed in this research, technical measures define the dimensions of a design space within both frameworks. Constraints limit a design space to acceptable designs while an objective function identifies preferred designs within such a design space. Figure 1 illustrates how technical measures can be illustrated for both constraints and objective functions in a two-dimensional design space. The two frameworks fundamentally differ on whether designers seek to *optimize* or *satisfice* in systems design (Guo et al. 2024; Vermillion and Malak 2020). Figure 1 (left) shows that any design that satisfies four constraints is acceptable. Figure 1 (right) shows an objective function that has value increasing as *measure a* and *measure b* increase. While the objective function framework can provide a ranking for system alternatives, the constraint framework can provide a determination of acceptability.

It is worth nothing that the two frameworks can be used together (*NASA Systems Engineering Handbook*, 2019; Vrolijk and Szajnfarber 2023), but will be evaluated comparatively in this research (Abdelli et al. 2020). Together, constraints can limit a design space with the most critical design needs or feasible alternatives. In parallel, an objective function can be used within that constrained design space to select a preferred alternative or aid in informing a preferred system design. Each framework offers benefits and challenges in the systems design process.

Normative and descriptive models fundamentally differ by stating what alternative should be selected, and what alternative will be selected. Sensitivity analysis and comparative tests can be performed to benchmark model behavior to real-world data. If provided real-world human selection data, descriptive models can use such data for validation, as it is what descriptive models seek to mimic. However, this data should not be used by normative models as they do not seek to mimic humans but seek to maximize value. Normative frameworks that seek to aid in design or decision making cannot be easily validated as discussed by (Chen et al. 2007).

#### Constraint framework as applied in practice

Requirements define an engineering problem (Buede and Miller 2024; Li et al. 2023; Ryan and Wheatcraft 2017), either mathematically or through sets of natural language statements which describe desires or limitations on a design. While requirements engineering is an entire field of study, the premise of a traditional requirements engineering process is that every requirement must be satisfied for a design to be acceptable. Requirements typically are derived from stakeholders or informed by physical interfaces, environmental limitations, or legacy knowledge (Murugaiyan et al. 2016). Organizations such as NASA treat technical measures as the dimensions, or “units” for requirements, either developing a set of technical measures prior to requirements or in conjunction (*NASA Systems Engineering Handbook*, 2019). Prior research has treated requirements as constraints (Murugaiyan et al. 2016).

#### Objective function framework as applied in practice

Multi-attribute decision making has been studied under multiple techniques, such as weighted sum, weighted product, etc. (Yeh 2002). As systems involve multiple stakeholders, disciplines, and subsystems, evaluating multiple, competing attributes together in a decision has held much appeal for the systems design community. Approaches like Quality Function Deployment (QFD) seek to take multiple customer needs and translate those into usable engineering attributes or requirements (Chan and Wu 2002). Approaches using objective functions for system decision making seek to rank order alternatives by amalgamating multiple attributes (Clerkin and Mesmer 2019a, b; Lee and Paredis 2014; Subramanian et al. 2016; Topcu and Mesmer 2015). Approaches using objective functions have a diverse history of development and application under terms such as Decision-Based Engineering (DBE) and Decision-Based Design (Chen et al. 2013; Mistree 1990; Mistree and Allen 1997; Mistree and Munster 1900; Wassenaar and Chen 2003), Value-Based Engineering (VBE) (Kis et al. 2018; Murugaiyan et al. 2016), Value-Based Design (VBD) (Bhatia and Mesmer 2017; Kannan et al. 2017), Value-Centric Design (VCD) (Brown et al. 2009; Keeney 1996; Richardson et al. 2012), and Value-Driven Design (VDD) (Cheung et al. 2012; Collopy and Hollingsworth 2009; Keller and Collopy 2013; Soban et al. 2012). Organizations including NASA have previously employed objective functions in the development of systems to inform decision making (*NASA Systems Engineering Handbook*, 2019). An objective function for systems design typically represents both the stakeholder preferences (Richardson et al. 2012) and the system itself, with no constraints or minimal constraints (Murugaiyan et al. 2016). While accurate and precise determination of the value of various system alternatives would be ideal, so long as the objective function can tell a decision maker which alternatives would be more preferred than others, decisions can be made. It has been stated that the goal of systems design is to create value (Lavi and Reich 2024).

#### Prior research on omission of measures

While prior research has not examined the impact of technical measurement in system acquisition processes, prior work in different domains has explored measure omission.

Omitted Variable Bias or Left Out Variable Error (OVB/LOVE) can occur in scenarios when a relevant (meaning impactful on the outcome) variable that is correlated with another relevant but included variable is not included in a model. The included variable’s impact on the outcome can then be incorrect, or biased due to misattributing the impact of the omitted variable. OVB/LOVE were developed in the field of psychology where criterion variables, such as *salary*, are desired to be understood by explanatory variables such as *age* or *education*. OVB/LOVE occurs in certain situations where an omitted variable has a significant effect on the criterion variable and is correlated to an included explanatory variable (Mauro 1990). Additional work has been done to examine the impacts of omissions from causal to latent variables and amongst different model levels and structures (Kim and Frees 2006; Sackett et al. 2003).

A few approaches to model omission can be observed under the umbrella of mathematical modeling for the objective function and multi-criteria decision-making problem domains. First, sensitivity analysis focuses on the impacts of changing various factors in a model including parameters, variables, initial conditions, choices of model configurations, etc. (Razavi et al. 2021). Sensitivity analysis has been explored in multi-criteria decision analysis (Triantaphyllou and Sánchez 1997). Efforts have been made to develop processes for decision making with uncertain data (Ma et al. 2013). Second, purposeful omission can be observed under model parsimony, where the simplest satisfactory model is desired.

Prior work has discussed the importance of completeness and consistency in requirements (Ferrari et al. 2014; Kannan 2025; Luitel et al. 2024; Zowghi and Gervasi 2002). Additionally, work exists in developing different development techniques to elicit a complete list of requirements from stakeholders (Pacheco et al. 2018; Zowghi and Coulin 2005; Zowghi and Gervasi 2002).

It is important to note that omission of measures is not the same as missing data points. Missing data points within a dimension requires different approaches for data analysis and modeling. The omission of a measure indicates that the corresponding dimension or concern is not considered. While in an ideal world, the set of technical measures considered would be the identical set of technical measures that are theoretically relevant to the decision makers, in practice the sets cannot be assumed to be the same, as discussed in Sect.  1.2.7 and 1.2.8.

#### Omitted technical measures are likely in systems engineering and design

It is often recommended that technical measure sets should be “complete”, or not miss any relevant measures (Bullock and Deckro 2006; Goh 2015; Mackley 2005; Sahraoui 2013; Staron 2012; Vanek et al. 2008; Verries et al. 2008). Ideal recommendations for technical measures are not always achieved in real world practice. Technical measures can be omitted for practical concerns, simplicity, bias, or oversight. The INCOSE Systems Engineering Measurement Primer instructs that “only the most critical parameters should be selected” due to costs (Roedler and Jones 2005). Regarding one type of technical measures, Measures of Effectiveness (MOEs), it is suggested “to list as many MOEs as possible, reserving the right to omit irrelevant or less important ones later.” (Pinker et al. 1995). When omitting measures using this guidance, it may not be clear how to determine which measures are irrelevant. While theoretically including all relevant technical measures would provide the *most information* to decision makers, in practice, (1) there must be a limit to the number of technical measures in a set, (2) it is often unknown what technical measures a complete or correct set should contain, and (3) more information may not lead to improved decisions. These limitations in practice create a scenario where the set of technical measures may be difficult to select and may differ from an idealized set.

In practice, the set of technical measures must be limited. Tracking technical measures can be costly (Expanded Guidance for NASA Systems Engineering. Volume 1, 2016), therefore practitioners are often instructed to select “only the most critical parameters” (Systems Engineering Measurement Primer v2.0: A Basic Introduction to Measurement Concepts and Use for Systems Engineering, 2010). Recommendations for “simplicity” (Collopy, 2009) in objective function formation can be found along with specifications of 10–20 attributes (considered technical measures in this research) (Collopy and Hollingsworth 2011). A recommendation in Expanded Guidance for NASA Systems Engineering suggests removing a requirement “when the cost of implementing the requirement adds more risk to the project by diverting resources than the risk of not complying with the requirement”(*Expanded Guidance for NASA Systems Engineering. Volume 1*, 2016). It has been observed that “real-world engineering decisions resist full quantification” (Ferguson & Bryden, 2026).

Guidance that can inform technical measure selection is limited (Eaton et al. 2024). Simply increasing the number of technical measures may not be a solution. Since technical measures interact as a set, a higher number of measures may result in no feasible design or increased complexity of analyses, potentially encouraging practitioners to select fewer measures. Beyond cost and complexity, more information may not result in improved decisions in practice. Prior design research has suggested that reducing variables considered may result in improved designs through providing “slack” (Vrolijk and Szajnfarber 2023). Reducing constraints to open the design space has been observed to only be able to result in improved alternative selection (Hazelrigg and Saari 2021). Adding information through decomposition increases problem complexity (Topcu et al. 2021). Increased information can increase the number of objectives or constraints designs must meet, potentially obfuscating the critical needs for a system. When considering human cognition, a high volume of information has been observed to negatively impact decision quality through mechanisms such as stress or cognitive overload (Phillips-Wren and Adya 2020). Research in other domains suggests that increased information does not necessarily lead to improved decisions (Todd 2007), particularly for experts (Ettenson et al. 1987). A balance must exist, understanding the trade-offs between quantity and costs of technical measures.

If it were simple to select a compete set, investigation into omission would be unnecessary. However, it is not simple to select a complete set, nor even assess what a complete set means. Therefore, we cannot approach technical measure sets assuming they are known to be complete. Similarly, the selection challenge could be overlooked if the results from the frameworks using the technical measures were easily validated. However, for the selection and design of large-scale, complex engineered systems, technical measures are used to aid decision making rather than mimic human decision making. For novel systems, little or no data may exist to benchmark both technical measures or resulting decisions. These challenges with technical measure selection suggest that technical measure sets should not be assumed to be complete in practice.

#### Direct observations of omitted technical measures

Omissions of technical measures can also be directly observed in prior research of the two frameworks. In value modeling efforts, research can be observed that examines a larger set of technical measures than is included, potentially omitting relevant measures. While it is possible that these removed technical measures could have been irrelevant, prior research indicates their removal can be caused by a practical limitation such funding or time constraints or oversight, rather a purposeful step in the theoretical framework development process. It has been observed that decision models are *often incomplete*, due to incapability of capturing all relevant requirements of a design (Guo and Chen 2024).

A 2021 paper developing value models for the NASA Human Landing System identifies 10 attributes applicable to the model but excludes 3 attributes due to difficulty in measuring them (Eaton and White 2021). A 2017 paper developing a value model for NASA funding allocations identifies additional preferences beyond those that were included in the objective function, indicating relevant technical measures were omitted in their model (Dyas and Mesmer 2017). A value model of a small satellite acknowledged that the developed model was simple and future models would expand the model to additional, relevant considerations (Bhatia and Mesmer 2017). When developing value models based on different perspectives, a 2018 paper identified example attributes for each model, acknowledging additional attributes exist (Bhatia et al. 2018). A value model of commercial aerospace systems omits qualitative attributes because they are difficult to quantify (Cheung et al. 2012). Beyond these examples where the omissions are acknowledged, it is also possible that measures are omitted unknowingly.

Selecting an ideal number of technical measures should consider the tradeoff between knowledge and costs incurred by tracking and computation. However, pressures of cost and simplicity, along with the difficulty of determining if a measure is relevant and a set of technical measures is complete, may lead to omitting measures that change the engineering decision. This research seeks to illustrate the impact of omissions of technical measures so that more attention to the tradeoffs may occur in practice.

### Research gap and contributions

Within systems acquisition and development, current gaps in technical measure selection guidance leave practitioners dependent on best practices and information from previous systems to inform technical measure selection as observed by (Eaton et al. 2022, 2024; Eggstaff et al. 2014; Pinker et al. 1995; Rhodes et al. 2009; Sillitto 2004). As technical measures direct the system alternative selection or system design decisions (Green 2001; Sillitto 2004), an incomplete set of technical measures can have significant impacts (Mackley 2005). System outcomes depend on formulating the correct problem as observed in prior research (Sigurdarson et al. 2025). This research highlights the challenges with problem formulation in the selection of technical measure sets.

This research provides case study evidence illustrating how omitting a technical measure can lead to design and/or system alternative selection decisions that differ from decisions that would be made if that technical measure was included. This research proposes that a set of technical measures can be a source of inconsistency in decision making if desired measures (from one or more stakeholders) are not included in (one or more) decision makers’ set of technical measures.

While some prior research has performed sensitivity analyses in different techniques within multi-criteria decision analysis (see (Demir et al. 2024) for a review), this research compares how the two frameworks rank and determine acceptability for system alternatives with omissions of technical measures in a systems acquisition case study. While the case study models a specific space system, the methodology can be applied to large-scale complex systems from other domains.

## Methodology

**Fig. 2:**
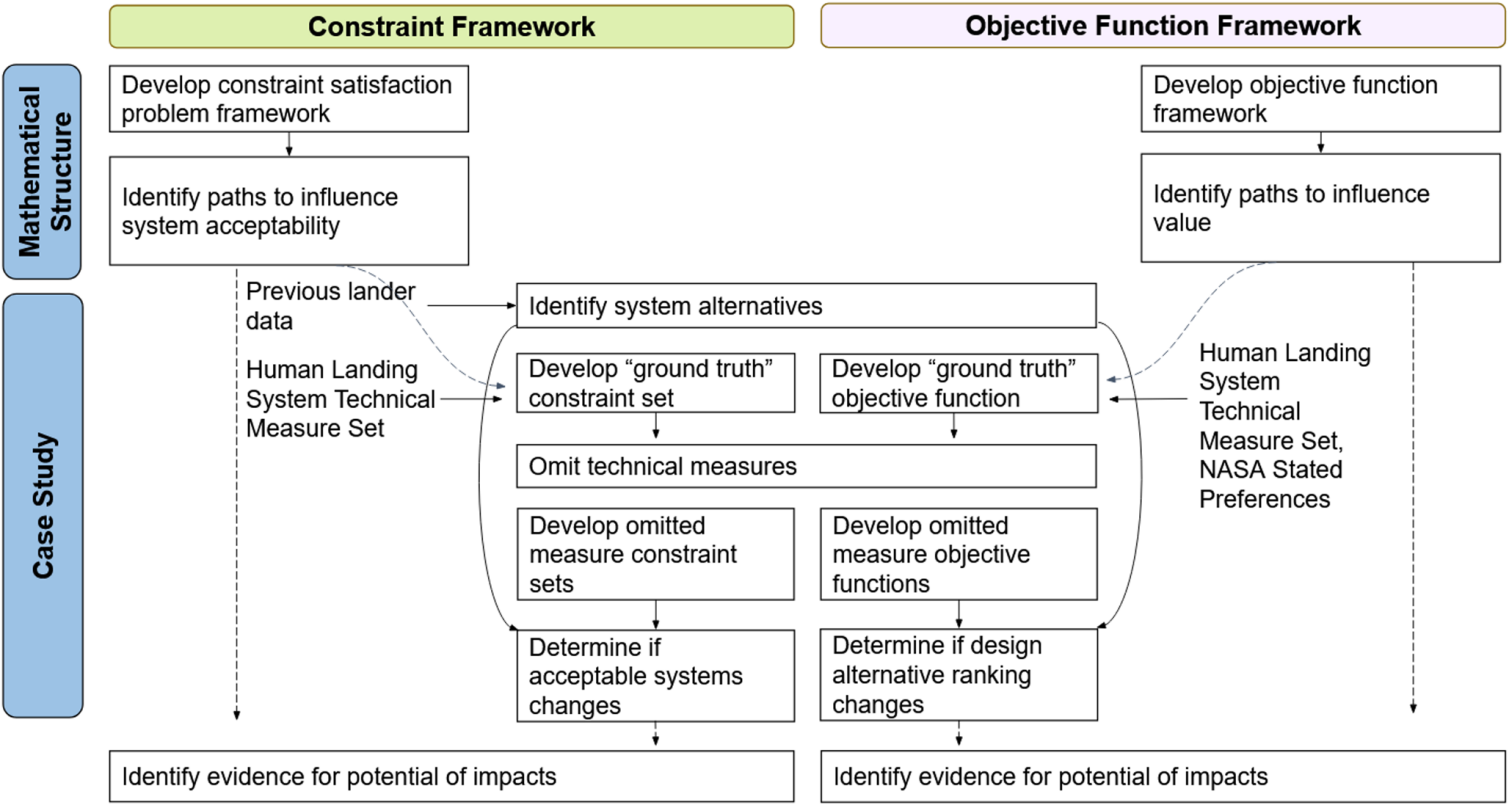
Methodology overview

Two frameworks (constraint and objective function) will be considered when assessing the impact of omitted measures. For the case study, one type of technical measure, technical performance measures will be used. Figure 2 provides an overview of the methodology that is detailed in the following sections.

### Mathematical structure for omission impacts

#### Developing a constraint framework mathematical structure

The impact of an omitted measure in a constraint framework on the acceptability of a system will be examined through a constraint satisfaction problem (CSP). Technical measures ($\:{m}_{1},\:{m}_{2},\:....\:{m}_{n})$ will be represented as constraints ($\:{g}_{j},{h}_{k})$ in a design space, where are $\:{g}_{j}$ are inequality constraints and $\:{h}_{k}$ are equality constraints, shown in Eqs. 1 and 2. Omission of technical measures will be represented as removing a unary constraint or removing that measure in a binary or higher order constraint.

1 $$\:{g}_{j}\left(m\right)\:\le\:\:0,\:j=1,..p\:$$

2 $$\:{h}_{k}\left(m\right)\:=\:0,\:k=1,..l\:$$

Constraints may be unary, binary, or higher order, depending on how many technical measures appear in a constraint. Constraints that are unary consist of one measure. Constraints that are binary or higher order consist of two or more measures. While technical measures in constraint frameworks can be unary, higher-level constraints often have measures with relationships to other measures. For example, total mass is related to multiple subsystem masses, even if it is not acknowledged explicitly through a constraint.

#### Developing an objective function framework mathematical structure

The impact of an omitted measure in a constraint framework on the value of a system will be examined mathematically through a value model. Technical measures will be represented as attributes ($\:{m}_{1},\:{m}_{2},\:....\:{m}_{n})$ in the weighted sum value model as shown in Eq. 3.

3 $$\:{\:v}_{i}={\beta\:}_{1}{m}_{i1}+{\beta\:}_{2}{m}_{i2}+...\:+{\beta\:}_{p}{m}_{xp}\:$$

Omission of technical measures will be represented by omitting the corresponding attributes from the value model.

### Developing the case study

A case study set of technical measures from a real-world large-scale, complex engineered system is also used to evaluate the impact of omitted measures.

#### Large-scale complex engineered system case study

The NASA Human Landing System (HLS) was selected to demonstrate the case study. The NASA HLS is large-scale, complex engineered system with multiple system alternatives considered and a publicly available technical measure set, making it suitable for this assessment of the impact of technical measure omissions. In 2020, a Broad Agency Announcement for the development of the HLS was released (*Next Space Technologies for Exploration Partnerships − 2 (NextSTEP-2) Appendix E: Human Landing System Studies*,* Risk Reduction*,* Development*,* and Demonstration*, 2019), resulting in multiple system alternatives developed in response to the announcement. In 2020, design development bids were awarded to SpaceX, Blue Origin, and Dynetics. In 2021 SpaceX was awarded a development contract for Starship. In 2023, a second development contract was awarded to Blue Origin.

#### Selecting technical measures for the case study

The set of technical measures for the HLS from HLS-PLAN-008-ANX03 will be treated as the “ground truth” set of technical measures. The measures will be grouped to represent high level similarities for clarity, though different groupings could be made.

Non-limiting measures are excluded, such as return propulsion mass, which is inherently less than its launch mass. Measures will also be excluded if no data is available for any previous systems. Definitions for the technical measures, both natural language and mathematical, will be identified from NASA documentation.

#### Estimations for incomplete data

Technical measures with the status “to be specified” per HLS-PLAN-008-ANX03 for goals/and or thresholds will be estimated. Measures specified to differ based on the provider will also be estimated. Estimations will be based on previous lander data and discussion with subject matter experts at NASA.

#### Identifying system alternatives

System alternatives are needed to evaluate how omitted measures can impact the system alternative chosen. In the constraint framework, how many system alternatives are acceptable according to the CSP with and without the omitted measure(s) will be assessed. In the objective function framework, the ranking of the system alternatives will be compared in the objective functions with and without the omitted measures.

Ideally, the actual system alternatives submitted for the HLS contract would serve as the system alternatives. However, only three HLS designs had publicly available data (Blue Moon, Starship, and ALPACA). Two proposed HLS designs by Boeing and Vivace will be excluded due to no data for any technical measures being publicly available, which may be due to early exclusion in the selection process. The National Team Integrated Lander Vehicle (ILV) National Human Landing System (NHLS) (proposed earlier in the contract process by Blue Origin, along with Lockheed Martin, Northrop Grumman, and Draper Laboratory) will also be excluded due to lack of available data.

To increase the number of system alternatives, prior human lunar landers were also added as system alternatives. Two were identified: the Lunar Module (LM) from the Apollo program and the Altair Lander concept from the Constellation program. These systems were included as they have the same purpose of transporting humans to and from the lunar surface.

To further increase the number of system alternatives, prior Mars landers were also added as system alternatives. While the HLS currently is planned for lunar landings, Artemis’ ultimate goal ends with human travel to Mars. A GAO report as of November 2023 stated that “The human landing system will provide crew access to the lunar surface and demonstrate initial capabilities required for deep space missions. SpaceX is currently developing a commercial Starship vehicle to transport humans and cargo to low-Earth orbit, the moon, and Mars” (Government Accountability Office, 2023). The HLS being identified as key infrastructure to enable future Mars landings makes the prior Mars landers of additional interest to include. Including both Mars and lunar landers will enable better assessment of impacts of omission on a larger set of alternatives. For the nine unmanned systems, it will be assumed that those systems would meet the crew requirement for the HLS for the sake of the case study. While this is not an accurate assumption, the technical measures identified do not explicitly include measures regarding the crew. Further, the research will not suggest that any of the system alternatives (whether actual bids, prior systems, or Mars landers) should be selected. The research is limited to examining the impact of omissions and the larger set of system alternatives better shows the impacts. A database of 3 proposed designs for the HLS, 2 previous NASA human landing systems or prototypes, and 10 other non-crewed NASA landing systems, will serve as the system alternatives. HLS alternatives include the Blue Moon Lander (Blue Origin); Starship (SpaceX); and Autonomous Logistics Platform for All-Moon Cargo Access (ALPACA) (Dynetics). Prior HLS designs include: the LM-1 (Apollo 5) and Altair (Constellation). *Prior non-crewed landing system designs* include Mighty Eagle (prototype); Morpheus (prototype); Insight Lander; Mars 2020 Entry, Descent and Landing System (EDLS); Mars Science Laboratory EDL; Phoenix; Mars Polar Lander; Viking 1; and Pathfinder.

Data for system alternatives will be identified from publicly available NASA documentation for each lander, including design reviews, press releases, fact sheets, program result reports, and budgets. Note that the identified data may have inaccuracies compared to the final designs due to some of the documentation being published before the designs were finalized. Data will be informed by subject matter experts when data for a technical measure is not available for a previous system.

#### Identifying and modeling preferences and system goals

NASA stated it wishes to “create a Human Landing System (HLS) to deliver human crews to the surface of the Moon” (*Next Space Technologies for Exploration Partnerships − 2 (NextSTEP-2) Appendix E: Human Landing System Studies*,* Risk Reduction*,* Development*,* and Demonstration*, 2019). This specific system goal was framed within the larger system goal of improving deep space exploration capabilities and stimulating the commercial space industry (*Next Space Technologies for Exploration Partnerships − 2 (NextSTEP-2)*, 2019). NASA stated they consider eight principles in their decisions: Fiscal realism, Scientific exploration, Technology pull and push, Gradual build up of capability, Economic opportunity, Architecture openness and resilience, Global collaboration and leadership, and Continuity of human space flight (*Next Space Technologies for Exploration Partnerships − 2 (NextSTEP-2)*, 2019). These stated principles, objective, and system goal provide a general structure for NASA’s preferences in developing the objective function and the direction for constraints when unclear from HLS documentation.

#### Developing the constraint framework case study constraint satisfaction problem

For technical measures with a threshold and goal, the constraints will be limited to the threshold with the goal indicating the feasible side of the constraint. For the measures with only a threshold, subject matter experts with familiarity with NASA space systems will be consulted on the direction. If a measure’s threshold equals its goal, it will be considered as forming an equality constraint. If the threshold and goal differed, a measure will be considered as forming an inequality constraint. Based on the definition for the measure, measures may compose unary, binary, or higher order constraints.

Which system alternatives are deemed acceptable will be compared between the full CSP with no omissions and the CSPs with omitted measures. In application it must be established how to deal with violations of constraints within a CSP. While CSP solutions can be complex, large-scale complex engineered system acquisition may not require advanced solving methods as they become more a verification process rather than a solution process. Two interpretations of a constraint framework will be explored: an interpretation where one measure not achieving its target is no different from all measures not achieving their targets and an interpretation where a design is “penalized” for each measure that does not achieve its target. The reported practice of rejecting a system if at least one technical measure fails to achieve its target (representable in Boolean algebra) will be compared with a more lenient approach of considering a system with one violated technical measure as “better” than a system with many violations. This comparison reflects both reported practice and hypothesized actual practice, where a few or minor violations may be addressed with a waiver or the offending constraint modified if the rest of a design is satisfactory.

#### Developing the objective function framework case study value model

Measures are included in the objective function, representing the preferences discussed in Sect.  2.2.5. The preferences for the HLS are identified in the protest of non-selection for awards by Dynetics and Blue Origin in 2021 which details the decision-making process. According to the GAO report, the technical approach had the highest impact on value, followed by price, followed by management approach (U. S. Government Accountability Office, 2021a). The details of the evaluation factors are shown in the Appendix in Table A1. Thus, the objective function will include technical measures to capture the technical approach factor, cost, and management factor.

Ideally the “weights” scaling the measures would be evidence-based conversion factors converting the inputs into a common value unit. For this study, conversion factors were sought from publicly available information to convert the measures to units of dollars (such as cost per kilogram for space launch). Most technical measures did not have this information. For these measures lacking information, the conversion factors were based on engineering knowledge from the authors who have a diverse engineering background. To ensure this resulted in a value model that aligned with NASA’s preference, the median measurement values were obtained from the system alternatives and the portions of the value model related to the technical approach, price, and management approach were calculated using these medians. These value model portions were observed to be highest for technical approach, followed by price, followed by management approach, mirroring the preferences stated by NASA. Since this research is only looking at the impact of omissions, a perfect value model is not required. The value model developed is merely an example that could represent one of many preferences of NASA. The developed value model will affect the specific outcomes from the case study. However, the research focuses on demonstrating that impacts can occur and describing the mechanisms in which omissions can occur and impacts propagate, rather than characterizing the specific omissions and their impacts specific system alternatives. Prior research acknowledges the difficulty in determining relative importance of attributes (Sigurdarson et al. 2025) and this research does not seek to improve upon this challenge in forming objective functions. The ranking of the system alternatives will be compared to identify if there are changes in the ranking of the system alternatives when measures are omitted. In other words, the ordinal consistency of the multiple value models will be examined.

#### Expectations for the frameworks based on real-world outcomes for the HLS

The outcomes of the two frameworks can be established based on the contract award decisions. For the constraint framework it would be expected that SpaceX’s Starship and Blue Origin’s Blue Moon systems would be acceptable due to being awarded contracts. Dynetics’ ALPACA would be anticipated to be unacceptable due to not being awarded the contract. For the objective function, it would be expected that SpaceX’s Starship would be preferred to Blue Origin’s Blue Moon which would be preferred to Dynetics’ ALPACA, due to the two-step contract award where SpaceX was selected to be a provider first followed by Blue Moon. While the 2021 NASA Selection Statement by Kathryn L. Lueders states no “comparative analysis or trade-off amongst proposals” (*Source Selection Statement: Appendix H: Human Landing System*,* Option A Next Space Technologies for Exploration Partnerships-2 (NextSTEP-2) NNH19ZCQ001K_APPENDIX-H-HLS*, 2021) were conducted, we suggest logically some preference ordering of the system proposals occurred, whether formalized or unstated, to first select SpaceX as a provider before selecting Blue Origin.

## Results

The mathematical structures for both frameworks will be modeled, followed by the case study findings.

### Mathematical structure for constraint framework

Equations 4 and 5 show two interpretations of constraint frameworks. Equation 4 shows a strict interpretation where if a constraint is not satisfied, the acceptability (P) of the system is a function of all measures 1 through n. If one measure is 0, indicating unacceptable, the entire design is 0, indicating unacceptable. Equation 5 shows an example of a penalty approach, where the CSP is transformed into a pseudo-objective function that minimizes the violation of constraints. For Eq. 5, the violations of the constraints are transformed into a common unit of value ($) by the conversion factor $$\:{w}_{j}$$. Equation 5 can be interpreted as representing the difficulty of a violated constraint. This overage is represented by a penalty from Eq. 5. For example, it can represent a cost associated with correcting a system design. For example, a launch weight may be limited by the weight a launch pad can support. If the launch weight exceeds this constraint, there is a cost incurred to change the design of the launch pad or develop a new one. Alternatively, it can represent a cost associated with procuring a waiver for that requirement. Though Eq. 5 does not model this scenario, in practice, if cost becomes prohibitive a project may be cancelled.

4 $$\:P\:=\:{m}_{1}\cdot\:{m}_{2}\:\cdot...\:{m}_{n\:}\:$$

5 $$min \:P(\overline{m})={\sum}_{j=1}^{p}\:max(0,{g}_{j}\left(\overline{m}\right)*{w}_{j})$$

It can be shown that omission of a measure in a constraint framework with a strict interpretation, such as Eq. 4, can change system acceptability. Take for a simple example two systems, System A and System B, which are each a function of three measures, but the third measure is omitted. If the third measure is violated in System A, but not System B, the systems would incorrectly label both systems as acceptable.

Equation 5 calculates a penalization associated with constraints that are not satisfied, potentially akin to requesting a waiver in systems design. The acceptability (P) is a function of measures ($\:{m}_{1},\:{m}_{2},\:....\:{m}_{n})$ indicating how many and to what extent constraints are violated.

It can be shown that omission of a measure in a constraint framework with a penalty interpretation, such as Eq. 5, can change system acceptability. Take for a simple example two systems, System A and System B, which are each a function of three measures (unary constraints), but the third measure is omitted. Measure 1 is not violated for either system. Measure 2 is minorly violated for System B. Measure 3 is severely violated for System A. With the complete set of measures, assuming the measures are of equal importance, System B should be chosen. With the omitted measure set, System A would be chosen.

### Mathematical structure for objective function framework

**Fig. 3:**
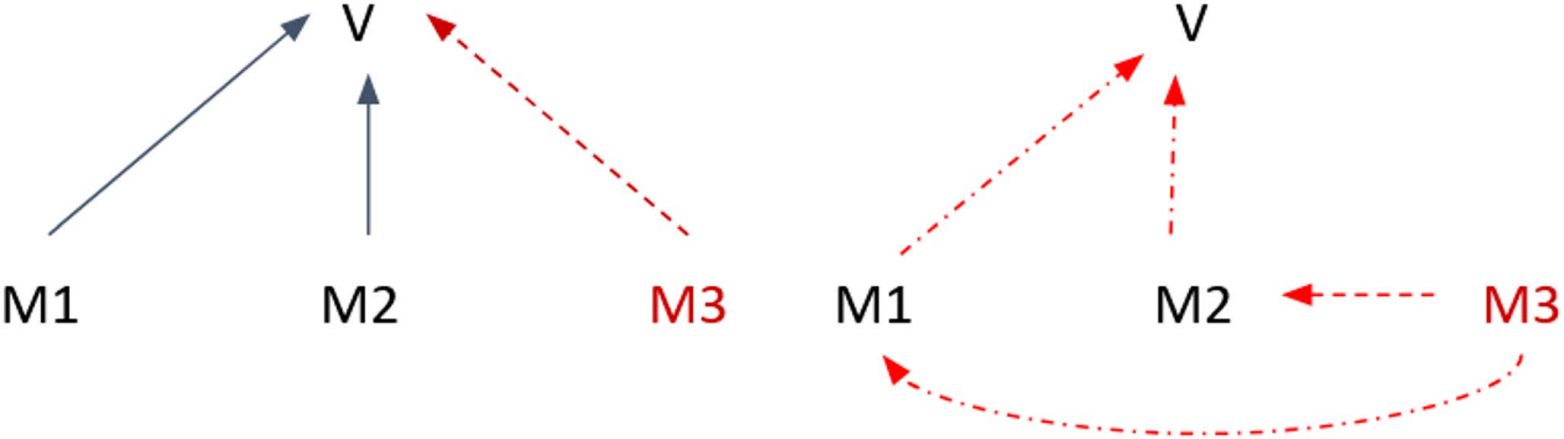
Technical measures direct and indirect connection to value

If measures are not coupled, the impact on value can be assessed directly as shown on the left for Fig. 3. For omissions where measures are coupled, indirect impacts through other measures also exist as shown on the right for Fig. 3. The impact on value can be written as Eq. 6:

6 $$\begin{aligned}\:dV/d{M}_{3}&=(\partial\:V/\partial\:{M}_{1})d{M}_{1}/d{M}_{3}\\&\quad+(\partial\:V/\partial\:{M}_{2})d{M}_{2}/d{M}_{3}\:+\partial\:V/\partial\:{M}_{3}\:\end{aligned}$$

### Case study results

The HLS case study consists of the system being mathematically modeled in the constraint framework and the objective function framework. The framework models are then tested by omitting varying measures and assessing the impacts on the system selected and the other included measures.

#### Case study measure estimations

53 measures were listed in the HLS Technical Measure Plan. 13 measures had accessible data for the system alternatives. Only 7 out of the 13 measures had complete data for all of the system alternatives: *Landing Accuracy (LA)*,* Specific Impulse (ISP)*,* Thrust (TP)*,* Usable Propellant Mass at Earth Launch (UPM)*,* Cargo Mass Capability (CMC)*,* Dry Mass at Earth Launch (DM)*,* and Launch Mass Allowable (LMA).*

For systems with gaps for the remaining 6 measures, the measures were estimated. The estimation methods depended on the technical measure as follows: *Communication Link Margin (CLM)* - mean of identified values from other systems; *Uncrewed Lunar Orbit Operations Duration (ULD)* - estimated to be the threshold for the technical measure; *Surface Mission Duration* (SMD) - estimated to be the threshold for the technical measure; *Lunar Darkness Survival Duration* (LDD) − 150 h; *Vehicle Reliability (VR)* − 0.98; *Slope Tolerance Maximum (ST)* - mean of identified values from other systems.

#### Constraint framework

13 unary inequality constraints, 13 non-negativity constraints, and 1 higher order inequality constraint were developed according to the required thresholds for the technical measures. The non-negativity constraints are non-restricting on the set of system alternatives. The complete constraint set can be found in the Appendix.

**Fig. 4:**
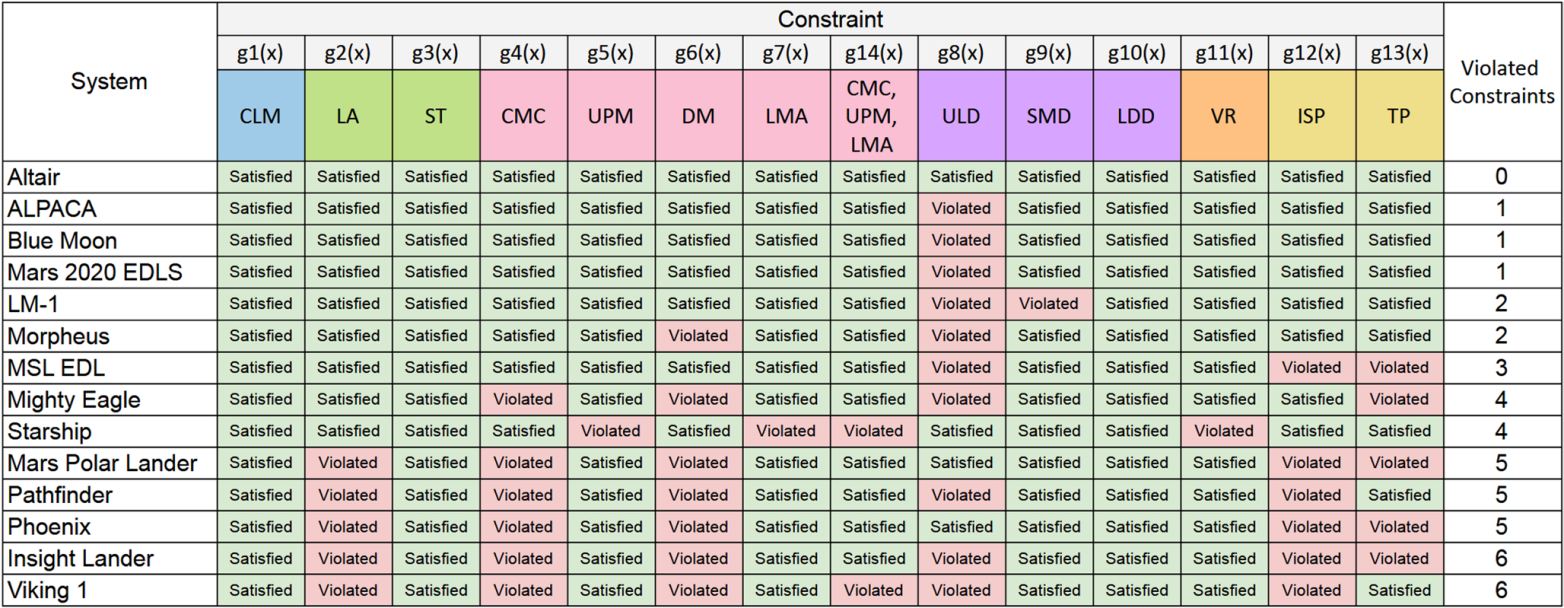
Strict interpretation: acceptable and unacceptable designs for complete measure set

Acceptable designs are those that do not violate any constraints for a strict interpretation. Figure 4 shows only one system alternative, the Altair Lander from the constellation era does not violate any constraints in the complete set of constraints, shown with a 0 in the rightmost column. The remaining system alternatives violate 1–6 constraints. Constraints that are satisfied are color coded in green; constraints that are violated are color coded in red. Measures are grouped by type and color coded for clarity amongst figures (e.g. mass-related measures are color coded in red).

**Fig. 5:**
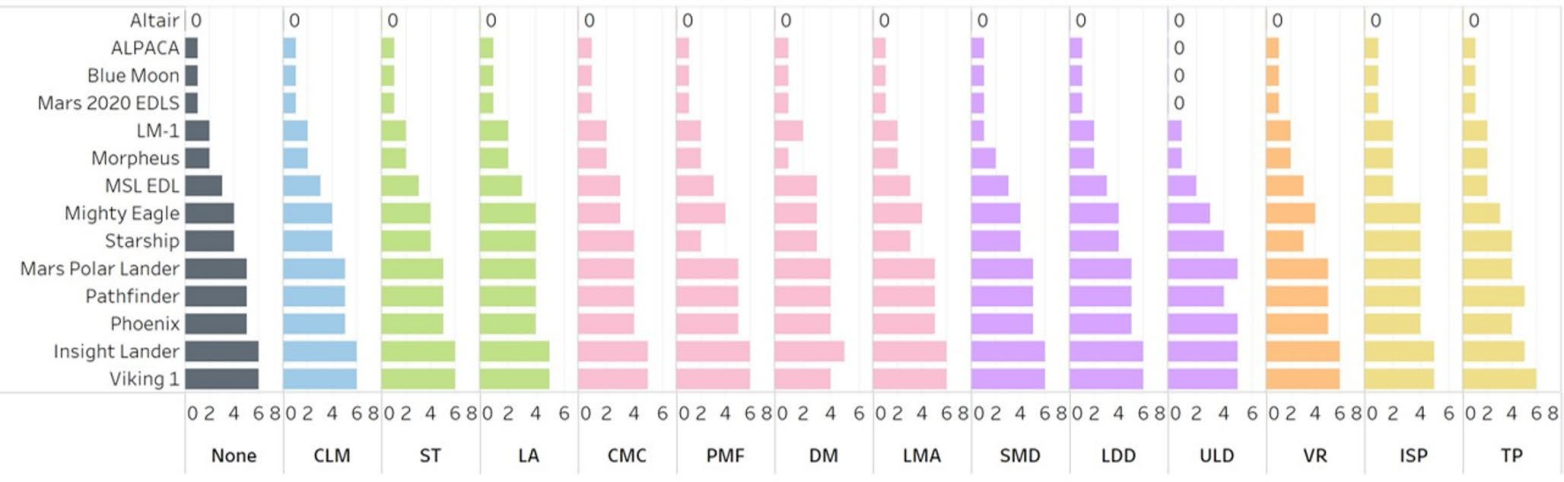
Strict interpretation: acceptable and unacceptable designs for omitted measure sets

Figure 5 indicates how many constraints are violated in different sets of constraints. The leftmost set of constraints shown in gray represents no omitted constraints and is essentially another representation of the last column in Fig. 4. The remaining columns show the number of violated constraints when specific measures are omitted. For example, the second column represents the number of violated constraints when the *Communication Link Margin* measure is omitted.

In this case study, only one measure’s omission, Uncrewed Lunar Orbit Duration, makes additional systems (3 systems in this case) acceptable when strictly interpreting the constraints. The omission of the remaining measures removes 1–2 violated constraints, but violated constraints still exist.

**Fig. 6:**
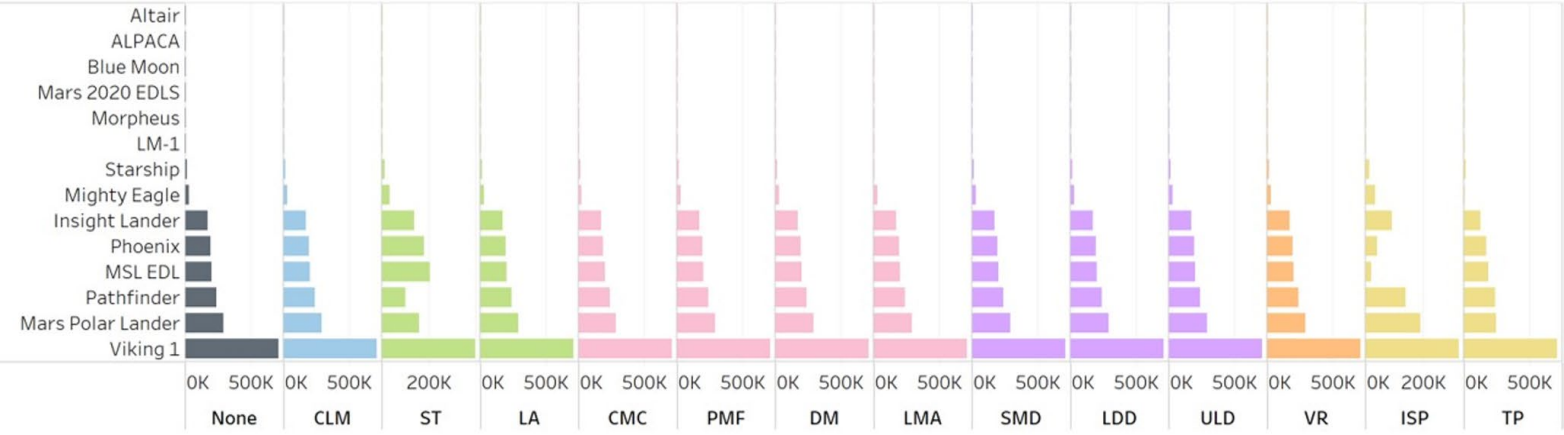
Penalty interpretation for omitted measure sets in constraint framework

Technical measures are most often evaluated with margins, meaning they are often not treated as the strict interpretation. For example, minor violations can often be managed with a waiver. A penalty interpretation of the constraints can be created, as shown in Fig. 6, with the measures weighted in the same process as followed in the objective function framework. A higher penalty indicates greater violations of constraints.

Figure 6 shows how some systems’ violations of one or more constraints were by a small amount, resulting in small penalties. Figures 5 and 6 indicate different acceptable systems. If “ranked” by the number of violated constraints and the extent of the penalty, the systems differ by the strict and penalty interpretations with the complete set of measures, as shown in the grey, leftmost columns in Figs. 5 and 6. The penalty interpretation makes 7 additional systems “acceptable” as compared to the strict interpretation if selected based on relatively small penalties with the complete set of measures. When omitting Specific Impulse, the penalty interpretation identifies two more systems as acceptable. (Phoenix and Mars Science Laboratory).

#### Objective function framework

**Fig. 7:**
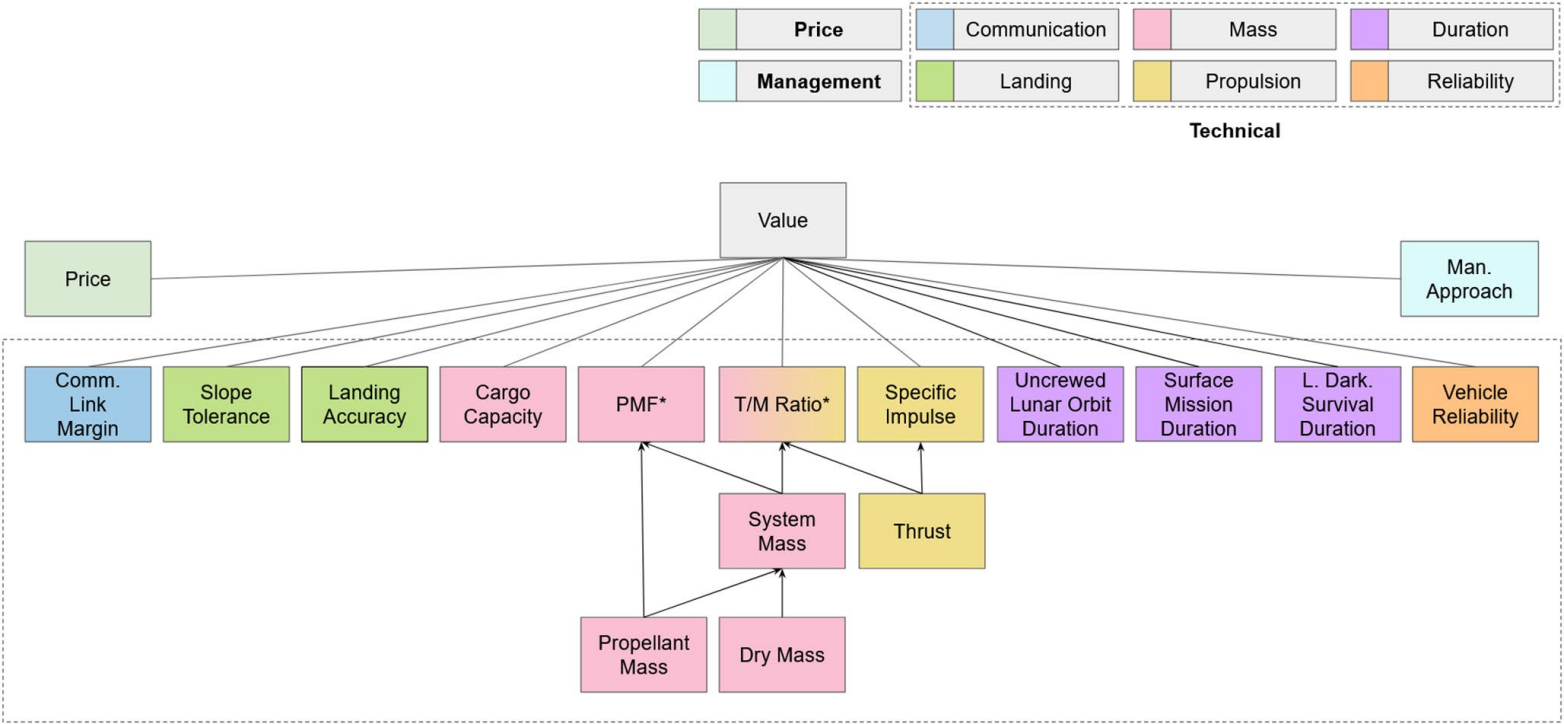
Graphical objective function

The technical measures used in the constraint framework, described in Sect.  3.2.1, appear as attributes in the value model objective function, representing the *technical* decision making evaluation factor, along with *price* and *management approach*, shown in Table 1. The management portion of the value model was not broken down into lower levels but represented with the evaluation factor *management approach*. 13 technical measures appear in the objective function framework case study, comprising 11 of the 13 technical measures. 4 of the 13 technical measures comprise 2 higher level technical measures which are noted with an asterisk: PMF and T: M Ratio, as shown in Fig. 7.

**Table 1:** Summary of technical measures and evaluation factors

Evaluation Factor	Measure Category	Technical Measure	Operationalized Definition (units)
**Total Evaluated Price**	*N/A*	Price (P)	The cost for the lander, adjusted for inflation to 2023 dollars (million $)
**Management Approach**	*N/A*	Management (M)	Five point scale assessing quality (5 -outstanding, 4 - very good, 3 - acceptable, 2 - marginal, 1- unsatisfactory) (unitless)
**Technical Approach**	*Communication*	Communication Link Margin (CLM)	The difference between the available signal- to-noise ratio and the signal-to-noise ratio required to achieve a level of reliability in terms of bit error ratio (dB)
	*Landing*	Slope Tolerance (ST)	The maximum ground slope that the vehicle is able to tolerate during landing (degrees)
		Landing accuracy (LA)	The distance from the true landing location to the planned, target landing site (m)
	*Mass*	*Propellant Mass Fraction (PMF)	Ratio between the propellant mass and initial mass of the HLS (unitless)
		Cargo Mass Capability (CMC)	The payload capacity to the lunar surface, including crew (kg)
		Dry Mass (DM)	The total dry hardware mass, excluding propellant and cargo mass (kg)
		Usable Propellant Mass at Earth Launch (UPM)	The total usable propellant including any reserve propellant, bias amounts, and estimated excess propellant at end of mission (kg)
		Launch Mass Allowable (LMA)	The limit to the total mass at Earth launch, including the launch vehicle adapter mass, cargo mass, and propellant mass (kg).
	*Mass/ Propulsion*	*Thrust to Mass Ratio (TM)	Thrust capability divided by the mass of the of the HLS (N/kg)
	*Propulsion*	Specific Impulse (ISP)	How efficiently the main HLS engines generate thrust (s)
		Thrust (TP)	Force produced by the main HLS engines (N)
	*Duration*	Uncrewed Lunar Orbit Duration (ULD)	Time the integrated lander is capable of remaining in a ready state in Lunar Orbit after completion of Lunar Orbit Checkout Review (LOCR) (days)
		Surface Mission Duration (SMD)	The duration that the vehicle is capable of being fully operational on the lunar surface after lunar landing and until ascent (days)
		Lunar Darkness Survival Duration (LDD)	The longest continuous lunar darkness period in which the vehicle can operate (hours)
	*Reliability*	Vehicle Reliability (VR)	System hardware reliability (without corrective repair) throughout the entirety of the defined mission (unitless)

**Table 2:** Evaluation factors for HLS decision making

Evaluation Factor	Areas of Focus
***Factor 1: Technical Approach***	*Technical Design Concept*
	*Development*,* Schedule*,* and Risk*
	*Verification*,* Validation*,* and Certification*
	*Insight*
	*Launch and Mission Operations*
	*Sustainability*
	*Approach to Early System Demonstrations*
***Factor 2: Total Evaluated Price***	*No focus areas*
***Factor 3: Management Approach***	*Organization and Management*
	*Schedule Management*
	*Risk Reduction*
	*Commercial Approach*
	*Base Period Performance*
	*Small Business Subcontracting Plan*
	*Data Rights*

For *management approach*, a prior system was labeled marginal if canceled, unsatisfactory if the system failed, acceptable for prototypes, and very good if implemented (see Table 1 for how *management approach* was operationalized). The technical measures (*technical* evaluation factor) plus the other two evaluation factors of *price* and *management approach* are arranged in an equation, shown in Eq. 7 and illustrated in Fig. 7 with units of millions of dollars.

7 $$\begin{aligned}\:V\:&=-\:P\:+\:300*M\:+\:300\$/dB*CLM\:+\:40\$/day*ULD\:\\&\quad+\:600\$/day\:*SMD\:+\:2\$/day*LDD\:\:\:+\:8000*VR\:\\&\quad-12\$/m*LA\:+\:50*ST\:\:+\:500\$/degrees*TM\:+\:{10,000}*\mathrm{PMF}\:\\&\quad+0.006\$/\mathrm{kg*CMC}\end{aligned}$$

Figure 7 shows the technical measures, color coded by measure category. The two technical measures composed from lower level technical measures are marked with an asterisk.

**Fig. 8:**
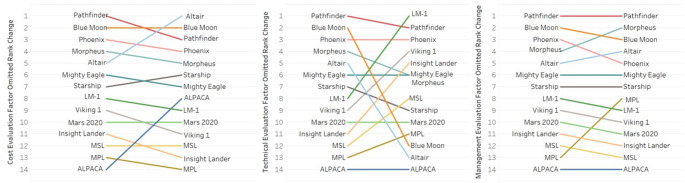
Change in rank for cost, technical, and management evaluation factor omission

Figure 8 shows the change in rank for the system alternatives when each of the three evaluation factors are omitted. Each evaluation factor changed the ranking of one or more systems. Kendall’s rank distance is a distance measure that assesses pairwise difference, where 0 would indicate no differences in rank and 1 when normalized would indicate an exact reversal of rankings. The ranking of the system is compared according to the objective function formed of all measures (no omissions) and the ranking of the objective functions formed from omissions. Kendall’s rank distance indicates there are differences in rank for cost (Kd = 0.166), management (Kd = 0.115), and technical evaluation factor omissions (Kd = 0.269), with omission of measures within the technical evaluation factor incurring the largest changes in rank.

**Fig. 9:**
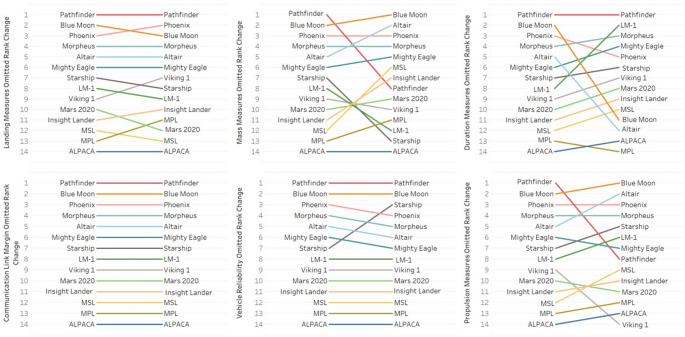
Change in rank for measure category omissions within the technical evaluation factor

Figure 9 shows omissions of measure categories within the technical evaluation factor. Every measure category omission changed the ranking of one or more system alternatives. Kendall’s rank distance indicates there are differences in rank when omitting landing measures (Kd = 0.077), mass measures (Kd = 0.295), propulsion measures (Kd = 0.218), and duration measures (Kd = 0.192).

**Fig. 10:**
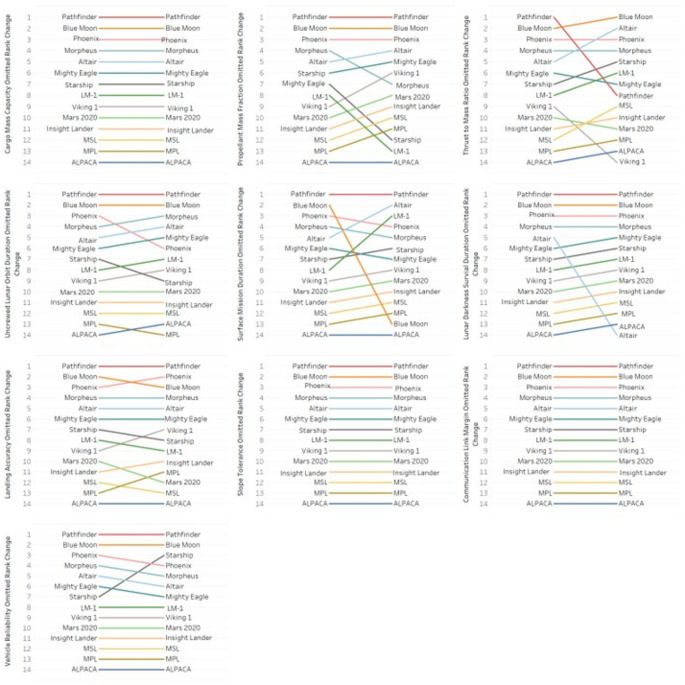
Change in rank for individual attribute omissions

Figure 10 shows omissions within the technical evaluation factor, omitting technical measures directly. Every technical measure omission changed the ranking of one or more system alternatives. Kendall’s rank distance indicates there are differences in rank when omitting Cargo Mass Capability (Kd = 0.026), Specific Impulse (ISP) (Kd = 0.013), Lunar Orbit Operations Duration (Kd = 0.218), Vehicle Reliability (Kd = 0.038), Surface Mission Duration (Kd = 0.641), Landing Accuracy (Kd = 0.077), T: M Ratio (Kd = 0.218), Slope Tolerance (Kd = 0.013), Lunar Darkness Survival Duration (Kd = 0.128), and Propellant Mass Fraction (PMF) (Kd = 0.141).

## Discussion

The cases in which omitting a measure changed the system alternative chosen are discussed in Sect.  4.1–4.2. The differences between the objective function framework and constraint framework are considered in Sect.  4.3. Implications from the research are discussed in Sect.  4.4–4.5. Limitations the work should be considered under are presented in Sect.  4.6.

### Constraint framework impacts

The research demonstrates that omitting a technical measure can change a system from being considered unacceptable to being considered acceptable if that measure appears in the limiting constraint(s), meaning active constraints, for an alternative. An inactive constraint does not change acceptability and can be considered “unnecessary” according to (Hazelrigg and Stolfi 2021). Similarly, constraints reduce the design space, and potentially exclude preferred alternatives as discussed by (Hazelrigg and Saari 2021). This research explored a system alternative selection problem where the alternatives are discrete. In systems design the alternatives are being developed. Many combinations of design choices may result in extensive system alternatives to select from, rather than a few discrete, realizable alternatives. This system alternative selection problem examined how many discrete system alternatives were feasible across different technical measure omissions with the assumption each system is realizable. If examining omission impacts during design, what portion of a design space opens with an omitted measure could be assessed. The opening of the design space could potentially increasing the number of feasible system alternatives. However, it may be difficult to characterize in systems design how many realizable system alternatives may be affected by omitted measures. Not all areas of the design space necessarily represent equal realizable system alternatives.

The likelihood of impacts in a constraint framework depends on the constraints imposed on the system and the system alternatives. For example, a highly constrained system in which the system alternatives violate more than one constraint would not change the acceptable system alternatives if one measure was omitted. However, as shown in the research, multiple omissions increases the potential for impacts, as the design space becomes increasingly opened.

The demonstrated impacts of omitting a technical measure in a constraint framework highlight a challenge with technical measures. Requirements must first be established to enable assessing if a system satisfies the constraints. An analysis of the limiting, active constraints depends on those requirements being constructed and designers having an understanding of system alternatives. A system alternative selection problem as demonstrated in this research provides the requirements as information to organizations which design a system alternative targeted to satisfy the constraints. For novel systems, this process requires either upfront analysis to develop the set of technical measures and the constraints or flexibility to evolve the technical measures and constraints as development efforts continue and more information is known about the system alternatives.

### Objective function framework impacts

The research demonstrates that in an objective function framework, impacts can occur from omitting technical measures. Similarly to the constraint framework, the likelihood and extent of the impact depends on the objective function and the system alternatives. An omitted technical measure must be “relevant”, meaning included in the objective function. Further, its impact must be relative to the other factors so as to cause a rank reversal. The type of technical measure and the weighting of the technical measures are inherently involved in the mechanism for omission impacts. For example, higher-level technical measures, through being aggregate of other technical measures and potentially relatively more heavily weighted in an objective function can affect the impact. Highly coupled technical measures can affect the impact through multiple paths.

In parallel, the system alternatives also determine the impact potential of omitting technical measure(s). For example, if system A is preferred across every technical measure over system B, omitting any number of technical measures up to n-1 measures will not change the ranking of system A and B. System alternatives which are highly disparate may be less sensitive to changes in omissions in the objective function because the changes must meet a greater magnitude to manifest in rank changes. Alternatively, if system alternatives are very similar, they may be more sensitive to impacts from omissions of technical measures in the objective function. For example, there would likely be a significant threshold required to reverse ranks when selecting between an automobile and a toy car for transportation, whereas that threshold is likely lower when selecting between a Honda CRV and a Honda HRV. While the degree of impact necessarily depends on the objective function formed and the system alternatives considered, this research shows that, when following common guidance and seeking to represent current practice, the objective function framework can be sensitive to omissions of technical measures.

### Comparing impacts between frameworks

In this case study, more inconsistencies in which system alternative should be chosen occurred in the objective function framework than the constraint framework. While the constraint and objective function frameworks are not directly comparable, the changes can be assessed. The acceptable system remained constant throughout all but one omission in the strict interpretation of the constraint framework. Comparatively, if the top ranked system is examined throughout the omissions in the objective function framework, four different systems were ranked first across the omissions (Pathfinder, Blue Moon, LM-1, and Altair). Inconsistencies in rankings appeared throughout the set of systems when comparing the complete measure set and omitted measure sets. This case study may support the idea that constraint frameworks may be more robust to measure omissions, however the generalizability of this observation may be limited to the specifics of the case study. This observation, though limited in generalizability, may be consistent with prior observations that satisficing strategies may be more robust than optimizing strategies to limited knowledge (Guo and Chen 2024). We do not suggest that omitting a technical measure will lead to a less preferred system being selected in either framework. It is possible a technical measure set may over-constrain a design space and an omission may result in selecting a more preferred system. However, it is important to note that both frameworks involve human input and assessments of their robustness to omissions cannot be fully characterized without considering the people engaging with the frameworks. Omissions in one framing, such as a constraint framework, may free a design space enabling better or more innovative designs or remove important considerations from being prioritized by a designer.

### Comparing actual award selections and model selections in the case study

The ideal systems determined in the analyses of this paper do not always align with the real-world selections by NASA of the SpaceX and Blue Origin systems. Inconsistencies with the actual decision are not unexpected. One source for the inconsistencies is that decision frameworks are observed to be imperfect (Collopy and Hollingsworth 2011; Eaton and White 2021; Lavi and Reich 2024). Another source for the inconsistencies is that real decisions are often viewed as imperfect. The NASA HLS initial award was contested. In April 2021, Dynetics and Blue Origin filed award protests to the Government Accountability Office (GAO) following the single award to SpaceX (Foust 2021), arguing in part that “NASA unreasonably evaluated all three of the proposals” (U. S. Government Accountability Office 2021b). The protests were denied in July 2021 (Cowing, 2021), though Blue Origin was later awarded a contract. This section discusses the inconsistencies to the real-world decisions, emphasizing areas of research needed for technical measurement.

Why were two systems (SpaceX’s Starship and Blue Origin’s Blue Moon) selected by NASA when they violated two constraints in the constraint framework? First, discrepancies may exist in the publicly available data for the systems and the data submitted to NASA, meaning there may not be actual violations. For example, the NASA Selection Statement regarding SpaceX claimed SpaceX’s design has substantially augmented capabilities, these do not come at the expense of heightened risk to mission execution or crew safety” but also stated “some development and technical risk necessarily accompany SpaceX’s innovative approach” (*Source Selection Statement: Appendix H: Human Landing System*,* Option A Next Space Technologies for Exploration Partnerships-2 (NextSTEP-2) NNH19ZCQ001K_APPENDIX-H-HLS*, 2021) For this research, the reliability data for SpaceX available from public documentation was relatively poor compared to other systems.

If there are no data discrepancies, one explanation may be that NASA may not use a constraint framework to award contracts. If NASA employs a constraint framework, it may be with a penalty interpretation. As discussed in the research, there may not always be a system alternative that meets every requirement. It is possible that the two selected systems were either preferred, and the constraints not considered critical. It is also possible that these two systems violated the fewest constraints, violated less critical constraints, or only minorly violated constraints, and were therefore selected. Iterative or flexible requirements, such as the penalty interpretation demonstrated, can enable this decision-making process along with waivers.

Straightforward and transparent frameworks provide less flexibility in decision making and present a risk of limiting the design space as observed in (Vrolijk and Szajnfarber 2023). In denying the award protests that occurred of the SpaceX award the GAO alludes to NASA maintaining decision control for the award: “The rules for these procurements are not the same as those for standard competitive federal procurements, as agencies generally enjoy broader discretion in selecting the proposals most suitable to meeting their research and development needs when utilizing broad agency announcement procedures.” (U. S. Government Accountability Office 2021b). Guidance for selecting sets of technical measures often suggests reuse of technical measures from prior similar systems (Eaton et al. 2024). The reuse may effectively result in a form of design fixation if the measures are associated with some level of architecture. Expert judgment or objective based approaches may be able to better reflect preferences than measure-based frameworks by avoiding unambiguous constraints.

Why did the three real-world bids (SpaceX’s Starship, Blue Origin’s Blue Moon, and Dynetics’ ALPACA) not maintain expected ordinal consistency in the objective function framework? This discrepancy can likely be attributed to our construction of the objective function. If NASA employed an objective function, it is likely that they would have used a different conversion scheme or formation method than used in this paper. There are many ways to develop an objective function and prior research has shown the formation method to have a significant impact on the ordinal consistency of value models (Eaton and White 2021). This research did not seek to rank the three systems according to NASA’s awards, but instead create an objective function based on the publicly available data that would have been available to the companies, including the measures and preference statements that appear in NASA documentation. Modification of the conversion factors could create an objective function that orders the systems according to the real-world decision, namely prioritizing cargo mass. The cargo capability of SpaceX’s system was acknowledged as “noteworthy” in exceeding NASA’s thresholds in the 2021 NASA Selection Statement from Kathryn L. Lueders (*Source Selection Statement: Appendix H: Human Landing System*,* Option A Next Space Technologies for Exploration Partnerships-2 (NextSTEP-2) NNH19ZCQ001K_APPENDIX-H-HLS*, 2021).

While the 2021 NASA Selection Statement proposes that they did not compare amongst proposals (*Source Selection Statement: Appendix H: Human Landing System*,* Option A Next Space Technologies for Exploration Partnerships-2 (NextSTEP-2) NNH19ZCQ001K_APPENDIX-H-HLS*, 2021), we suggest that ranking or preference must have occurred to choose one design prior to the second award. It is also likely that decision makers at NASA are influenced by unstated technical measures or non-technical aspects. For example, the NASA decision statement mentions SpaceX’s architecture, which is uncaptured in the technical measures.

The objective function and constraint frameworks can be used together. If combined in this case study, the Altair system would be chosen due to being the highest value system (ranked fourth) that satisfies all the constraints, as it was the only system that satisfied all the constraints. Different systems would be chosen according to each framework if used independently. An objective function framework alone indicates Pathfinder to be the preferred system. A constraint framework alone indicates that Altair is the only acceptable system.

### Limitations

The inconsistencies to the real-world award decision for the HLS are discussed in Sect.  4.4. The remaining limitations are discussed in this section.

#### System alternative limitations

We were unable to include all contract bids (excluding Boeing and Vivace) due to some designs having no publicly available data. This means only three bid designs for the HLS were analyzed. The system data was gathered from publicly available reports and websites. There may be differences in the publicly available documentation and direct proposal submissions to NASA. There is also potential that the descriptions of data in the sources may have evolved over time. This means while the name of a measure may stay the same across all landers, the actual quantity being measured may differ. For example, in some documentation (on the crewed landers), it was unclear if payload capacity included or excluded the crew.

Our dataset also included Mars landers, which clearly have design differences to lunar landers. The inclusion of the additional Mars landers is justified by the HLS being part of the architecture for both lunar and Mars landings (Government Accountability Office, 2023). The most obvious example of the differences of the Mars landers is that no Mars landers were crewed. While the prior Mars landers were not crewed, the set of technical measures examined did not include technical measures tied explicitly to the presence of a crew, such as pressurized volume, etc. The Mars systems make more apparent the inconsistencies that can result from omissions of technical measures, rather than serve as valid alternatives. The research does not suggest actually selecting a prior Mars lander for the HLS, but shows that with a pool of potential system alternatives, inconsistent decisions can be made between a complete and omitted measure set. Not all reported technical measures from NASA documentation were able to be included in the research, due to availability of gathering those measures for the systems. The omissions of these technical measures may have an impact on the reported acceptable/unacceptable and preferred systems shown in this research. The impact would appear in those measures being considered in the frameworks, similarly to how the initial starting set was considered “complete”; a new larger starting set could then be considered “complete”. The purpose of the research is not to select a HLS system alternative, but to show how omissions can impact the system alternative chosen.

#### Framework development limitations

The strict interpretation CSP is developed directly from the NASA thresholds and goals. It is possible that the estimated thresholds are not accurate to those used by the companies bidding. For the penalty interpretation CSP, many different penalty functions or weightings could be developed. The example shown in this research is not intended to be interpreted as the “correct” method for creating a penalty function. This research focuses on providing evidence that omitting technical measures can impact the system alternative chosen, rather than determining if an awarded system should have been chosen.

There are many approaches to forming objective functions and an objective function itself is only one of many ways to model a multi-criteria decision making problem. There are many ways to weight multiple measures within a value model and current practice relies on the expertise of the designer to make these choices (Sigurdarson et al. 2025). This research does not claim the value model presented in this research is the best or only way to model preferences for the HLS. The development of the value model followed practice in literature and serves to provide evidence that omissions in technical measures can impact the system alternative chosen.

## Conclusions

This research established that in practice, we should not expect complete sets of technical measures, based on observations that measure sets are incomplete in research and likely to be incomplete according to current practice. When selecting measures, it is likely that systems designers may not be able to select the ideal set of measures. Little work has established what an ideal set means or even what a complete set means. Little guidance exists instructing how to select sets of technical measures. Technical measures must be selected before all desired data is available. Technical measures can be costly to gather and track. Further, changing stakeholders or changes in preferences may impact the desired set of technical measures, mirroring an omission.

Motivated by the observations of omitted measures, the lack of formal theory for selection of technical measures, and the practical constraints on selecting a theoretically complete set of measures, the research demonstrated that the incomplete sets can impact the resulting system. While this result is expected, the impacts of omitted measures, or even differing sets of measures, often go unacknowledged in constraint and objective function frameworks. Work on problem formulation and more specifically, technical measure selection, often does not accompany framework development for system alternative selection. Given the difficulty to formulate and validate design problems, this work seeks to emphasize the need for greater connection between problem formulation, solving, and validation. Progress in problem solving approaches in either a constraint framework or objective function framework must consider problem formulation and validation. More broadly, this work demonstrates the impact of human and the necessarily subjective nature to system alternative selection, even with the use of technical measures.

This research suggests that a constraint framework may be more robust to omissions. This is consistent with prior suggestions that satisficing strategies in design may require less knowledge than optimizing strategies (Guo and Chen 2024). However, this observation is limited in generalizability. More broadly, the research did not examine omission impacts in light of human behavior. How selection approaches are influenced by human behavior and how selection approaches and framing influence human choice remain as open questions.

Within the frameworks, omitting technical measures has the potential to change what system alternative is chosen. Regardless of framework, the resulting system is dependent upon the set of technical measures selected during design, that is, the problem formulated. While this finding is not surprising, it is important.

### Future expansion of this research

While prior work has examined the sensitivity of multi-criteria decision-making methods, this research is the first to examine technical measure omission (1) in system acquisition contexts and (2) comparatively across objective function and constraint frameworks. Future work can expand the modeling in a few areas.

First, the case study dealt with technical performance measures, which are a common technical measure. Incorporating different types of technical measures, such as measures of performance, measures of effectiveness, or leading indicators, that are used in different ways and are at a different level in the hierarchy of measures will provide further insights on how hierarchical or dependent measures behave with omissions.

Second, this paper concludes that measures should be managed under the assumption of uncertainty in the set of technical measures themselves. The research can be expanded to evaluate variation in the measure thresholds to better assess the sensitivity of the results to changes that could propagate from changing stakeholder preferences across the system acquisition lifecycle.

Third, the case study evaluated 14 real-world landing systems. While these prior systems provide clear, discrete sample data, future work can expand into simulated systems to transform the case study from an *evaluation* problem to a *design* problem. Future work can evaluate the bounds of the omission impacts and better enable a combination of modeling constraints and objective functions together.

Finally, the research focused on the impacts that occur mathematically within a framework from omitted measures, comparing across two frameworks. However, the choice of framework has been shown to impact system outcomes through human cognition impacts. Future work can assess human cognition impacts from the two different frameworks when measures are omitted in addition to the approach-based impacts of omitted measures examined in this research.

### Contributions

This research demonstrated a gap between theory and practice for technical measure sets in system alternative selection problems. The research combined observations of omissions in practice for technical measure sets with a demonstration that impacts of omission can be meaningful. The observation of omissions justifies the investigation into the impacts; the demonstration of possibility of impacts from omissions points to a need to further investigate how people select technical measures for system alternative selection problems in practice. Despite being described as objective or consistent, technical measure sets in both constraint and objective function frameworks are necessarily difficult to form, verify, and validate when used for system alternative selection. Whereas in many decision-making scenarios, results can be benchmarked or validated against prior results or human decisions, system alternative selection problems can largely only be assessed after a system is selected and realized. That assessment is generally limited to opinions of stakeholders, as the chosen system alternative usually cannot be compared to the competing alternatives if they were not developed and fielded. Determining the success of a system can be a contested endeavor (Eaton et al. 2025). Technical measures can provide framing to aid in assessing system alternatives. However, technical measure selection remains subjective and difficult to validate.

The research demonstrated that frameworks that seek to provide objective, consistent information, when put into practice, can provide inconsistent results because of omissions in technical measures. It cannot be assumed that all *ideal* technical measures are included in technical measure sets *in practice*. Following this, it cannot be assumed that system alternative selection frameworks will always provide “objective” or “consistent” results, even when following current best practices.

As its major contribution, this research first (1) observed that the selection of technical measures was subject to omissions when put into practice. This research then (2) built upon the characterization of practice to demonstrate that the known and likely omissions can impact the outcome of these frameworks, albeit through different mechanisms if satisficing or optimizing.

## Appendix

*Communication*:

*Communication Link Margin:* ${g}_{1}\left(x\right)=-CLM+3\:\le\:\:0$  

$$\\\qquad\:0\le\:CLM$$Landing:

Landing Accuracy: $${g}_{2}\left(x\right)=LA-50\:\le\:\:0$$

Slope Tolerance: $${g}_{3}\left(x\right)=-ST+10\:\le\:\:0$$

 $$0\le\:LA$$

 $$0\le\:ST\:\le\:180$$

Mass:

Cargo Mass Capability: ${g}_{4}\left(x\right)=-CMC+1524\:\:\le\:\:0$

 Usable Propellant Mass at Earth Launch: ${g}_{5}\left(x\right)=UPM-\mathrm{92,185}\:\:\le\:\:0$

 Launch Mass Allowable: ${g}_{6}\left(x\right)=LMA-\mathrm{106,847}\le\:0$ 

Dry Mass at Earth Launch: ${g}_{7}\left(x\right)=-DM+\mathrm{20,605}\le\:0$ 

Mass Relationship: 

$${g}_{14}\left(x\right)=UPM\:+CMC-LMA\le\:0$$

 $$0\le\:CMC,\:UPM,\:LMA,\:DM$$

 Duration: 

Uncrewed Lunar Orbit Operations Duration: ${g}_{8}\left(x\right)=-ULD+90\le\:0$

 Surface Mission Duration: ${g}_{9}\left(x\right)=-SMD+6.5\le0$

Lunar Darkness Survival Duration:${g}_{10}\left(x\right)=-LDD+150\le\:\:0$

$$0\le\:ULD,\:SMD,\:LDD$$

 System:

Vehicle Reliability: $${g}_{11}\left(x\right)=-VR+.975\le\:\:0$$

$$0\le\:VR\:\le\:1$$ 

Propulsion:

Specific Impulse: $${g}_{12}\left(x\right)=-ISP+300\:\le\:\:0$$

 Thrust of Main Propulsion System: ${g}_{13}\left(x\right)=-TP+\:8000\le\:\:0$

 $$0\le\:ISP,\:TP$$ 

**Table A1:** Evaluation Factors for HLS Decision Making

Evaluation Factor	Areas of Focus
***Factor 1: Technical Approach***	*Technical Design Concept*
	*Development*,* Schedule*,* and Risk*
	*Verification*,* Validation*,* and Certification*
	*Insight*
	*Launch and Mission Operations*
	*Sustainability*
	*Approach to Early System Demonstrations*
***Factor 2: Total Evaluated Price***	*No focus areas*
***Factor 3: Management Approach***	*Organization and Management*
	*Schedule Management*
	*Risk Reduction*
	*Commercial Approach*
	*Base Period Performance*
	*Small Business Subcontracting Plan*
	*Data Rights*

## Data Availability

The data are available upon request and with the permission of NASA.
